# Sensitive and Selective Detection of Oxo-Form Organophosphorus Pesticides Based on CdSe/ZnS Quantum Dots

**DOI:** 10.3390/molecules22091421

**Published:** 2017-08-28

**Authors:** Jinchao Wei, Jiliang Cao, Hao Hu, Qing Yang, Fengqing Yang, Jianbo Wan, Huanxing Su, Chengwei He, Peng Li, Yitao Wang

**Affiliations:** 1 State Key Laboratory of Quality Research in Chinese Medicine, Institute of Chinese Medical Sciences, University of Macau, Macau 999078, China; wjc551@sina.com (J.W.); caojiliang126@126.com (J.C.); haohu_um@163.com (H.H.); wjbcpu@sina.com (J.W.); huanxinsu@yeah.net (H.S.); chengweihe038@163.com (C.H.); 2 State Key Laboratory of Hydraulics and Mountain River Engineering, Sichuan University, Chengdu 610000, Sichuan, China; yuan_jia_hu@126.com; 3 School of Chemistry and Chemical Engineering, Chongqing University, Chongqing 400015, China; fengqingyang_cq@126.com

**Keywords:** organophosphorus pesticide, quantum dots, acetylcholinesterase, biosensor, *Panax ginseng*

## Abstract

A rapid, sensitive and enzyme-based optical biosensor was applied for the determination of seven organophosphorus pesticides (OPPs), including the oxo forms (malaoxon, paraoxon, dibrom, and dichlorvos), the thio forms (malathion and parathion) and the mixed form (demeton) in *Panax ginseng*. The principal of the proposed method is that the fluorescence quenching effect of quantum dots (QDs) can be observed by enzyme-generated H_2_O_2_. The active centers of acetylcholinesterase (AChE) could be inhibited in the presence of pesticides, which caused decrease of the generated H_2_O_2_. Then, the inhibition efficiency of pesticide to AChE activity could be evaluated by measuring the fluorescence changes. Different from biosensors based on immobilized enzyme or self-assembling technique, the proposed biosensor demonstrated a good selectivity for the detection of oxo forms of OPPs. In the present study, the important experimental conditions of the proposed biosensor were investigated. Under the optimized conditions (incubation temperature, 35 °C; incubation time, 20 min; pH value, 8.0; detection time, 30 min; AChE concentration, 40.9 U/L; and choline oxidase (ChOx) concentration, 637.5 U/L), the limit of detection for the investigated oxo-form OPPs was no more than 0.05 μM, which suggested that the proposed method could be used for sensitive and selective determination of trace amounts of OPPs residues in real samples with complex matrices.

## 1. Introduction

The past decades have witnessed a significant rise in global agricultural production owing to the boom in human population. A myriad of pesticides, including organophosphates, organochlorine, carbamates and triazines, have been extensively used to kill or to control unwanted pests, insects or fungi in the process of food and herbal production [1,2]. Much research in recent years has been conducted on the organophosphorus pesticides (OPPs), which are the most commonly used insecticides due to their low cost, high efficiency for insect elimination and faster degradation in the environment [3]. As a representative of the acetylcholinesterase (AChE) activity inhibitors, OPPs could be transferred to humans via the food chain and then inhibit the cholinesterase from breaking down acetyl choline, which would result in irreversible effects on the nervous system [4,5,6,7]. Furthermore, the oncogenic and teratogenic risks caused by OPPs have been reported [8]. According to the different kinds of atoms bonded to the phosphorus atom via a double bond, OPPs can be classified into two groups: the oxo forms, with a terminal oxygen connected to phosphorus by a double bond (P=O); and the thio forms, with the double bonded oxygen replaced by sulfur (P=S). Although many OPPs are the inhibitors of AChE, the OPPs with a P=O structure show the higher toxicity on human or animals than those with P=S structure according to the investigation on the metabolism of pesticides [4,9]. Traditionally, the OPPs have been detected using chromatographic methods which involves liquid or gas chromatography coupled to electron capture detector (ECD), nitrogen phosphorus detector (NPD), flame photometric detector (FPD), or mass spectrometry (MS) detector [10,11,12]. Obviously, these techniques involve extraction of large volumes of organic solvent, time-consuming, require extensive purification, and demand expensive equipment. By contrast, sensing techniques are very attractive for developing rapid platforms for on-site monitoring [13]. Moreover, the rising concern of the food safety has led to the considerable interest in the development of a simple, rapid, sensitive and reliable assay for the detection of OPPs, especially those with P=O structure is very important for food safety monitoring.

Quantum dots (QDs) known as semiconductor nanocrystals have been widely studied and applied in the detection of trace amounts of hazardous substance including pesticide and heavy metals over the past decades. Firstly, a remarkable variety of biosensors based on core-only QDs have been developed with their particular electronic and optical properties [14,15,16,17,18,19,20,21,22,23]. The most attractive properties of QDs are broad absorption spectra, narrow and symmetric emission bands, less environmentally sensitive, and high quantum yield [24,25,26,27]. These advantages of QDs in the narrow emission band have enabled the sensitive detection of trace elements without spectral interferences. Comparing with core-only QDs, core/shell QDs show the advantages of higher resistance to chemical degradation and higher quantum yield because of the protection of a inorganic shell layer on the QD surface [28,29]. Consequently, most QDs-related biosensors reported so far have been developed based on core/shell QDs [30,31]. In recent years, there are a few reports on sensitive detection of OPPs by using the core/shell QDs-based biosensors. (CdSe)ZnS QDs coupling with the organophosphorus hydrolase (OPH) have been applied for the detection of para-oxon [32]. As an electrochemical sensor, ZnS@CdS QDs labeled monoclonal anti-AChE antibody were used to quantify the OPPs [33,34]. Later on, CdTe/CdS QDs with DNA aptamers have been fabricated for sensitive determination of four OPPs combined with capillary electrophoresis [35]. However, these biosensors rely on the complex and tedious immobilized or self-assembling procedure. Moreover, the applicability of the above biosensors was not investigated comprehensively since most of them selected para-oxon as model analyte.

**Figure 1:**
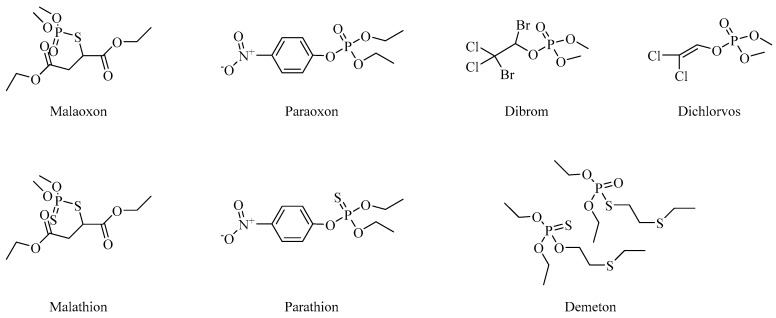
Structures of the studied pesticides.

In this study, an enzyme-based optical biosensor has been developed for the determination of seven OPPs, including malaoxon (**1**), paraoxon (**2**), dibrom (**3**), dichlorvos (**4**), malathion (**5**), parathion (**6**), and demeton (**7**) (Figure 1). Different from the aforementioned biosensors, the proposed solution-based biosensor provides a simple, rapid, and reliable approach without any complex assembly process. The principle of the method is the inhibition efficiency of pesticide to AChE activity could be evaluated by measuring the fluorescence changes of QDs. The systematic research on the different inhibition effects of oxo form and thio form OPPs has been performed. And the proposed biosensor showed good selectivity for the detection of oxo forms of OPPs with P=O structure. Because of the similarity in the inhibition effect between the proposed biosensor and the human body in the presence of OPPs, it may provide a new and powerful method for screening strong AChE inhibitors for the treatment of Alzheimer’s disease. Since the matrix effect often occurs in the analysis of real samples and influences the quantitation of the analytes according to the description in the European guidelines [36], it is essential to evaluate the applicability of the proposed method for the analysis of real samples with complex matrices. In this study, the developed method was applied to determine the investigated OPPs residues in *Panax ginseng*, a very famous Chinese herb. To the best of our knowledge, it is the first time the different inhibition effects of oxo form and thio form OPPs on AChE in real samples on the basis of core-shell QDs have been investigated.

## 2. Results and Discussion

### 2.1. Characterization of CdSe/ZnS Core/Shell QDs

The CdSe/ZnS core/shell QDs were optically characterized using UV-Vis absorption and fluorescence emission spectra. The absorption and emission spectra of the QDs in aqueous solution were presented in Figure S1. As shown in the figure, a narrow emission spectrum range from 550 to 650 nm was observed, which indicated that the size of the QDs was uniform. The fluorescence emission spectrum showed a peak centered at 597 nm upon excitation at 360 nm.

### 2.2. Principle of Sensitive Detection of Organophosphorus Pesticide

**Scheme 1:**
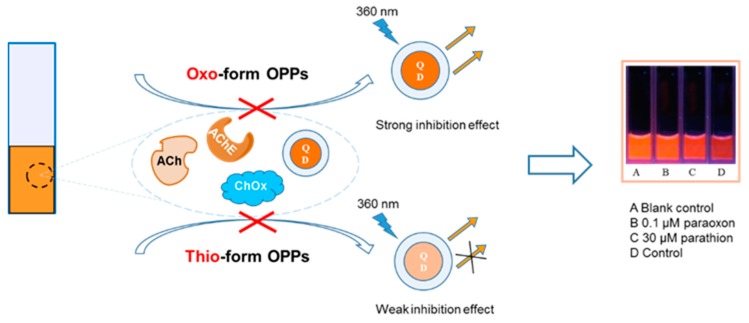
Schematic illustration of the biosensor for the detection of organophosphorus pesticides (OPPs).

The detection method in this study was based on principle that the AChE activity could be inhibited by OPPs. In comparison with the previous biosensors [33,34], the proposed method without immobilized or self-assembling procedure showed simple, reliable, good selectivity for the detection of OPPs residues. As illustrated in Scheme 1, ACh was firstly hydrolyzed to choline by AChE and then oxidized to betaine with the generation of H_2_O_2_ in the presence of ChOx. The fluorescence quenching effect of QDs could be observed in the presence of enzyme-generated H_2_O_2_. The active centers of AChE could be inhibited in the presence of pesticides, which caused the decrease of the generated H_2_O_2,_ and consequently the increase of fluorescence intensity of QDs. Therefore, the inhibition efficiency of pesticide to AChE activity could be evaluated by measuring the fluorescence changes. Previous studies have identified the close linkage between the inhibition efficiency and the pesticide concentration [18,37,38].

### 2.3. Optimization of Experimental Parameters

Several parameters of the detection procedure, including incubation temperature, incubation time, pH value, detection time, and the concentrations of AChE and ChOx were investigated in this experiment for the sensitive determination of pesticides.

Firstly, the effects of incubation temperature and incubation time on the fluorescence quenching in the presence of ACh were examined. The quenching efficiency (*∆F_t_*) values were calculated via Equation (1).

(1) $$\Delta F_{t}=\;F_{O}\;-\;F_{t}$$

where *F_o_* and *F_t_* are the fluorescence intensity recorded at 597 nm before and at different times after the addition of ACh in the detection system, respectively. According to the result, the value of the *∆F_t_* reached a maximum when the temperature reached 35 °C, which was in accordance to the optimum temperature of the catalytic behavior of enzymes [15]. While referring to the incubation time, the quenching efficiency reached a maximum and tended to plateau value when the incubation time was extended to 20 min. Moreover, the optimum pH values were 8.0–9.0 and 7.0–8.0 for AChE and ChOx, respectively. As a result, all the following experiments were carried out at pH 8.0. Consequently, the detection system was incubated at 35 °C for 20 min at pH 8.0 before fluorescence measurement in the further experiments.

The concentrations of both enzymes could affect the production of H_2_O_2_ and consequently have an influence on the fluorescence intensity. Firstly, the choline oxidation reaction catalyzed by ChOx was carried out based on diffusion controlled conditions. The different concentrations of ChOx from 2.0 to 637.5 U/L were analyzed in the study. After 425 μL of ChOx incubated for 20 min at 35 °C, 450 μL of the choline (440 μM) and 125 μL of the QDs (0.02 μM) were stepwise added into the ChOx solution and the fluorescence intensity was measured at 30 min (*F*_30_). As shown in Figure S2, there was a significant fluorescence changes (*∆F*) when the ChOx concentration was increased from 2.0 to 212.5 U/L. When the ChOx concentration was more than 212.5 U/L, a slight increment of fluorescence intensity could be obtained. Since sufficient amount of ChOx was one of the keys to the production of H_2_O_2_, 637.5 U/L was chosen as the optimum concentration of ChOx for the detection in the further experiment.

ACh hydrolysis reaction was performed under a kinetically controlled condition. In this study, the effect of AChE concentration within the range of 0.4−203.4 U/L on the fluorescence changes was investigated while the other parameters in this system remained constant. As shown in Figure S3, the fluorescence changes (*∆F*) of the QDs displayed obvious increase with the AChE concentrations increased from 0.4 to 40.8 U/L in the mixed solution. However, there was no remarkable increase in the fluorescence changes when the AChE concentration was more than 40.8 U/L. For sensitive detection of pesticides, a proper AChE concentration was normally required in this system. The low concentration of AChE could be inhibited by a small amount of pesticides, a narrow linear range between the inhibition efficiency and the pesticide concentration could be generated. On the contrary, a wide linear range could be obtained in the presence of the high concentration of AChE, whereas the trace amount of pesticides under the maximum residue limits (MRLs) may not be detected. Therefore, two AChE concentrations including 4.1 and 40.8 U/mL were investigated in the following study.

**Figure 2:**
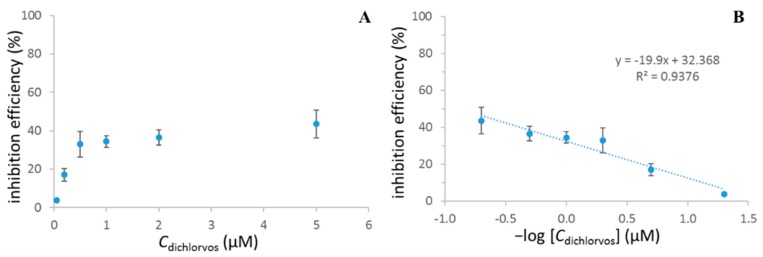
The plots of inhibition efficiency vs. dichlorvos concentration (**A**) and inhibition efficiency vs. the logarithm of dichlorvos concentration (**B**). The concentrations of QDs, acetylcholinesterase (AChE) and choline oxidase (ChOx) in all experiments were 2.5 nM, 40.8 U/mL and 637.5 U/mL, respectively.

In the present work, in order to evaluate the inhibition effect of pesticide to AChE activity, Dichlorvos was selected as the representative organophosphorus pesticide. The inhibition efficiency values (IE, %) were calculated according to the following Equation (2):

(2) $$\mathrm{IE}\;\left(\%\right)\;=\;\frac{\Delta F_{30\;without}\;-\;\Delta F_{30\;with}}{\Delta F_{30\;without}}\;\times \;100$$

where *∆F*_30 *without*_ and *∆F*_30 *with*_ are the absolute quenching efficiency for the first 30 min without inhibition and with inhibition at a certain concentration of pesticide, respectively. As illustrated in Figure 2A, the inhibition efficiency was increased significantly along with the increase of dichlorvos concentration from 0.05 to 0.5 μM in the presence of 40.8 U/L AChE. When the concentration of dichlorvos was further increased from 0.5 to 5 μM, only a slight increase of inhibition efficiency was observed. When the concentration values were transformed into the logarithmic form, the regression analysis between the IE values and the dichlorvos concentrations (ranged from 0.05 to 5 μM) displayed a satisfactory linearity with R^2^ value of 0.9376 (As presented in Figure 2B). On the other hand, when the concentration of AChE was 4.1 U/L, an acceptable linearity between the IE and the logarithm of the dichlorvos concentration could be obtained within a narrow concentration range of 0.001 to 0.01 μM. As a result, an AChE concentration of 40.8 U/L was chosen for pesticide detection. 

**Figure 3:**
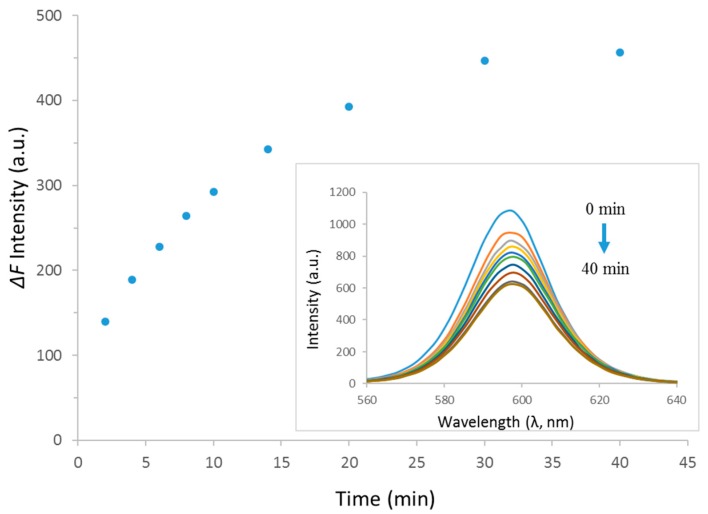
Typical time-dependent fluorescence changes of 2.5 nM quantum dots (QDs) in the presence of 637.5 U/L choline oxidase (ChOx), 40.8 U/L acetylcholinesterase (AChE) and 300 μM acetylcholine chloride (ACh): 2, 4, 6, 8, 10, 14, 20, 30 and 40 min. The inset displays plots of emission spectra of QDs.

In order to investigate the effect of the detection time for the bio-enzyme system on fluorescence changes, different times selected for the study were 2, 4, 6, 8, 10, 14, 20, 30 and 40 min (Figure 3). It was found that QDs fluorescence was quenched by the generated H_2_O_2_ and the fluorescence intensity started to show slight change after 30 min. As a result, 30 min was chosen as the optimum detection time for the further study.

### 2.4. The Reproducibility of the Proposed Biosensor

The reproducibility of the proposed biosensor was evaluated by comparing variations in fluorescence intensity on five consecutive days in the presence of 637.5 U/L ChOx, 40.8 U/L AChE and 300 μM ACh (Figure S4). The result showed that the relative standard deviation (RSD) of the quenching efficiency was 2.12%, which indicated the good reproducibility of the proposed biosensor.

### 2.5. The Anti-Interference Ability of the Proposed Biosensor

In order to evaluate the selectivity of this method for OPP detection, the representative coexistence substances were used for interference study. As shown in Figure S5, the addition of 10^4^-fold excess of Na^+^, Mg^2+^, glucose and alanine, 2 × 10^3^-fold excess of tyrosine on the determination of 1 × 10^−7^ M paraoxon did not cause any observable changes. Slightly interference effects were obtained after the addition of 10^4^-fold excess of K^+^ and sodium citrate. The above results proved the proposed method can provide credible anti-interference ability.

### 2.6. Inhibition Efficiencies of the Different Pesticides

In the previous study on the development of biosensor for the detection of OPPs, paraoxon was normally selected only [20,32,34]. In order to examine the applicability of this method comprehensively, both the oxo form and thio form OPPs, including dichlorvos and another six commonly used OPPs (paraoxon, parathion, malaoxon, malathion, dibrom and demeton), were investigated in this study. The matrix effect is the phenomenon caused by undetected components from the sample matrix, which will influence the linearity regress equation and quantitation of the analytes according to the description in the European guidelines [36]. It is necessary to evaluate the applicability of the proposed method for the analysis of real samples with complex matrices. The most widely used approach to overcome the matrix effect is the matrix-matched standard [6,39,40]. In this study, the developed method was applied to determine the investigated OPPs residues in *Panax ginseng*, a very famous Chinese herb. The linearity for all the studied pesticides was investigated in calibration standards prepared in *Panax ginseng* extract. As mentioned previously, the OPPs with the P=O structure possess a higher toxicity than the OPPs with the P=S structure. Thus, the oxo-form OPPs were expected to exhibit more efficient inhibitory effect on AChE activity than the thio forms. 

**Figure 4:**
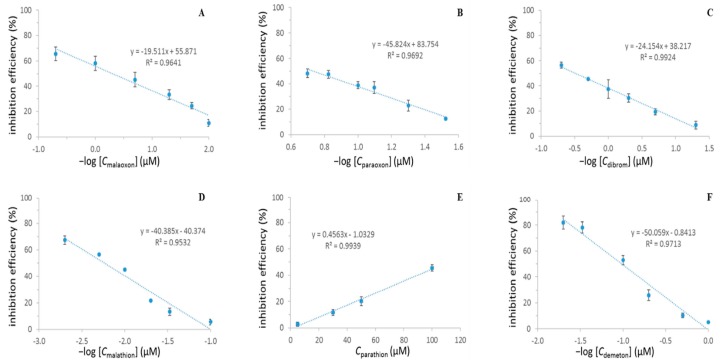
The plots of inhibition efficiency vs. the logarithm concentrations of malaoxon (**A**); paraoxon (**B**); dibrom (**C**); malathion (**D**) and demeton (**F**); and the plots of inhibition efficiency vs. the parathion (**E**) concentration. The concentrations of QDs, AChE and ChOx in all experiments were 2.5 nM, 40.8 U/mL and 637.5 U/mL, respectively.

As shown in Figure 4 and Figure S6, the oxo-form OPPs (paraoxon, malaoxon and dibrom), the thio-form OPPs (malathion and parathion) and the mixed form (demeton) exhibited good linearity between the inhibition efficiency and the logarithm of concentration. The lowest detectable concentrations (LDCs) for malaoxon, paraoxon and dibrom were 0.01, 0.03 and 0.05 μM, respectively, which were much lower than the MRLs regulated by the United States Food and Drug Administration (FDA) as well as European Union (EU). While in reference to the thio-form OPPs, the LDCs for malathion and parathion were 10 and 5 μM, respectively. As a mixture of isomeric forms, demeton consists of demeton-O (thiono isomer) and demeton-S (thiol isomer). It was observed that the LDC for demeton was 1 μM. Moreover, IC_20_, the concentration that induces an inhibitory effect of 20%, could be used to evaluate the strength of inhibitory effects of the different OPPs according to the US Environmental Protection Agency. In this study, the IC_20_ values were determined using the linear regression equations (see in Figure 4). Malaoxon, paraoxon and dibrom showed strong inhibitory effects with IC_20_ of 0.01, 0.04 and 0.18 μM, respectively. While the IC_20_ values of demeton, malathion and parathion were 2.61, 31.26 and 46.09 μM. These results confirmed that the oxo-form OPPs showed stronger inhibitory effect on AChE activity than the corresponding thio-form OPPs. The stronger inhibitory effect of oxo-form OPPs might result from the more electropositive phosphorus atom in the P=O linkage, which could facilitate the attack on phosphorus by the serine hydroxyl of AChE [4].

Recovery studies were performed at two spiking levels of the pesticide in real ginseng sample. As shown in Table S1, the recoveries were calculated to be 68.87–142.55%, and no pesticide has been detected in commercially ginseng sample. Both two batches of ginseng sample bought from herbal medicine market were not contaminated by the selected OPPs. Furthermore, the above result was confirmed by GC-MS while the experimental conditions were in accordance to the previous report [41].

## 3. Materials and Methods

### 3.1. Chemicals and Reagents

Acetylcholinesterase (Type VI-S, EC 3.1.1.7, from *Electrophorus electricus*, lyophilized powder, 217 U/mg) and choline oxidase (EC 1.1.3.17, from *Alcaligenes* sp., lyophilized powder, 15 U/mg), acetylcholine chloride (ACh), bovine serum albumin (BSA) and hydrogen peroxide (H_2_O_2_) were purchased from Sigma-Aldrich. Certified reference pesticide standards were all purchased from Dr. Ehrensdorfer (Augsburg, Germany). Water-soluble CdSe/ZnS core/shell quantum dots (QDs) were obtained from Ocean Nanotech (Springdale, AR, USA). Phosphate buffer solution (PBS) (pH 8.0, 10 mM) and Milli-Q ultrapure water (Millipore, ≥18 MΩ·cm) were used throughout. All chemicals were used without further purification. Individual stock solutions of the pesticides were prepared in acetonitrile and stored at 4 °C. A series of working solutions were prepared daily by an appropriate dilution by PBS (pH 8.0, 10 mM) which contains 0.5 mg/mL of BSA.

### 3.2. Apparatus and Software

The UV-Vis absorption spectra were measured using a Hach’s DR 6000 UV-Vis spectrophotometer. The fluorescence measurements were recorded on a Lumina fluorescence spectrometer (Thermo Fisher Scientific, Waltham, MA, USA). The exciting slit and the emission slit were both 5 nm. The samples for the fluorescence measurements were placed in a 10mm optical path length quartz fluorescence cuvette. Ultrasonic equipment (SCIENTZ SB-300 DTY, Ningbo Scientz Biotechnology Co., Ltd., Ningbo, China) was used for sample treatment.

### 3.3. Fluorescence Quenching Effect of QDs by Enzyme-Generated H_2_O_2_

According to the previous literature, ACh can be hydrolyzed and oxidized by AChE and choline oxidase (ChOx) with the generation of H_2_O_2_, and then fluorescence quenching effect of QDs can be obtained by enzyme-generated H_2_O_2_. AChE (136 U/L, 300 μL) was first incubated with PBS solution (10 mM, 100 μL) at 35 °C for 20 min. ChOx (1.5 U/mL, 425 μL), QDs (0.02 μM, 125 μL) and ACh (6 mM, 50 μL) were stepwise added in the above solution and the fluorescence signal was monitored over time. The fluorescence spectra of the sample were recorded in the emission wavelength range of 550–650 nm. The fluorescence intensity was measured at wavelength of 597 nm.

### 3.4. Procedure for the Sensitive Determination of Pesticides in Panax ginseng

Dried *Panax ginseng* material was purchased from herbal medicine market in Guangzhou, China. The sample pieces were cut into small slices about 2 mm in diameter and then mixed thoroughly. 1.0 g of each sample was weighted into a 15 mL glass centrifuge tube and soaked with 10 mL acetonitrile. The tube was then extracted for 10 min by ultrasonic water bath (10 min and 60 kHz) and centrifuged for 5 min at 800 g at 25 °C. 1.0 mL of the upper layer of the extract was transferred into a 2.0 mL centrifuged tube and evaporated to dryness under a stream of nitrogen. The residue was reconstituted in 1.0 mL of PBS for analysis.

Pesticides can inhibit the activity of AChE, and consequently induce a decrease in the fluorescence quenching efficiency of QDs by enzyme-generated H_2_O_2_. AChE (136 U/L, 300 μL) was first incubated with different concentrations of pesticides in *Panax ginseng* extract (100 μL) for 20 min at 35 °C. ChOx (1.5 U/mL, 425 μL), QDs (0.02 μM, 125 μL) and ACh (6 mM, 50 μL) were stepwise added in the mixed solution and the fluorescence signal was monitored over time. The fluorescence intensity at 597 nm was measured.

## 4. Conclusions

In summary, a rapid, sensitive and enzyme-based QD biosensor has been developed for the detection of organophosphorus pesticides. Since the oxo-form OPPs showed stronger inhibitory effect on AChE activity than the corresponding thio-form OPPs in this study, the proposed method seemed more suitable for the determination of the OPPs with a P=O structure. The limit of detection for all the investigated oxo-form OPPs, including dichlorvos, paraoxon, malaoxon and dibrom, were much lower than the MRLs regulated by FDA and EU. Unfortunately, it is difficult to admit that each of the developed methods based on AChE will has a specific problem. AChE is easily denatured by environmental change, and the detection results obtained by AChE based method are easily influenced by experimental conditions, such as pH value, incubation time, incubation temperature and so on. All in all, in comparison with biosensors based on the immobilized enzyme or self-assembling technique, the proposed biosensor showed a simple, reliable, good selectivity for the detection of oxo forms of OPP residues in real samples with complex matrices. Moreover, the similarity in the inhibition effect between the proposed biosensor and the human body may eventually lead to its application in strong AChE inhibitor screening.

## Supplementary Materials

Supplementary materials are available online.
