# Supplementary material for: Sensitive and Selective Detection of Oxo-Form Organophosphorus Pesticides Based on CdSe/ZnS Quantum Dots
Source: Molecules. 2017 Aug 28;22(9):1421. doi: 10.3390/molecules22091421 (PMC6151729; doi:10.3390/molecules22091421)
Supplement: Supplementary file 1 [file molecules-22-01421-s001.pdf]

Supplementary Materials

Sensitive and selective detection of oxo-form organophosphorus pesticides based on CdSe/ZnS quantum dots

Jinchao Wei [1], Jiliang Cao [1], Hao Hu [1,2], Qing Yang [2], Fengqing Yang [3], Jianbo Wan [1], Huanxing Su [1], Chengwei He [1], Peng Li [1,*], and Yitao Wang [1,]**

[1] State Key Laboratory of Quality Research in Chinese Medicine, Institute of Chinese Medical Sciences, University of Macau, Macau, China

[2] State Key Laboratory of Hydraulics and Mountain River Engineering, Sichuan University, Chengdu, Sichuan, China

[3] School of Chemistry and Chemical Engineering, Chongqing University, Chongqing, China

* State Key Laboratory of Quality Research in Chinese Medicine, Institute of Chinese Medical Sciences, University of Macau, Macau 999078, China

Correspondence: E-mail addresses: pli1978@hotmail.com (P. Li), ytwang@umac.mo (Y. Wang)

Figure captions

Fig.S1 The obvious UV-vis absorption peak and the fluorescence emission of the QDs with the maximum emission at 597nm.

Fig.S2 The fluorescence changes of 2.5 nM QDs with different concentration of ChOx (2.0, 8.0, 31.9, 63.8, 127.5, 212.5, 425.0, 637.5 U/L) in the presence of 200 μM choline; The inset displays plots of the fluorescence intensity changes of QDs solution versus the logarithm of ChOx concentration.

Fig.S3 The fluorescence changes of 2.5 nM QDs with different concentration of AChE (0.4, 1.3, 4.1, 8.6, 17.2, 40.8, 203.4 U/L) in the presence of 300 μM Ach, 637.5 U/L ChOx; The inset displays plots of the fluorescence intensity changes of QDs solution versus the logarithm of AChE concentration.

Fig.S4 The reproducibility of the proposed biosensor at five days

Fig.S5 The interference of coexisting substances on the detection of paraoxon.

Fig.S6 The plots of inhibition efficiency vs the Malaoxon (A), Paraoxon (B), Dibrom (C), Malathion (D), Parathion (E) and Demeton (F) concentration. The concentrations of QDs, AChE and ChOx in all experiments are 2.5 nM, 40.8 U/mL and 637.5 U/mL, respectively.

Fig.S1

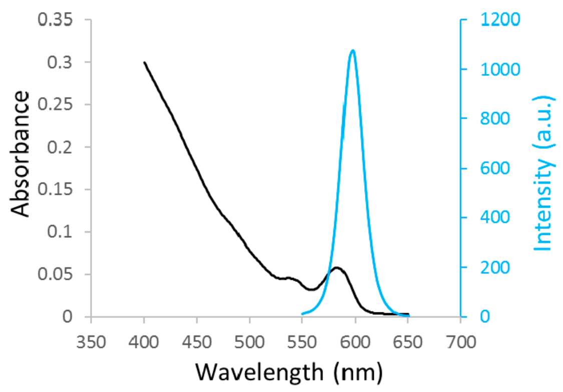

0.35
0.3
0.25
0.2
0.15
0.1
0.05
0
Absorbance
1200
1000
800
600
400
200
0
Intensity (a.u.)
350 400 450 500 550 600 650 700
Wavelength (nm)

Fig.S2

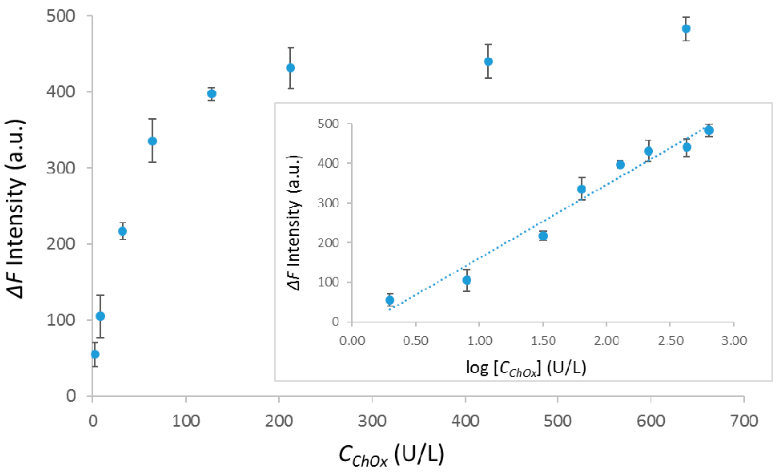

500
400
300
200
100
0
ΔF Intensity (a.u.)
0 100 200 300 400 500 600 700
C_{ChOx} (U/L)
ΔF Intensity (a.u.)
0.00 0.50 1.00 1.50 2.00 2.50 3.00
log [C_{ChOx}] (U/L)

Fig.S3

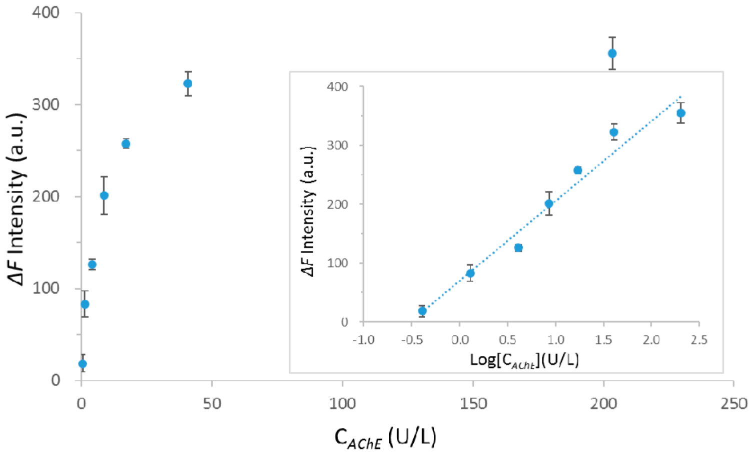
400
300
200
100
0
ΔF Intensity (a.u.)
0
50
100
150
200
250
C_AChE (U/L)
ΔF Intensity (a.u.)
Log[C_AChE](U/L)
-1.0
-0.5
0.0
0.5
1.0
1.5
2.0
2.5

Fig.S4

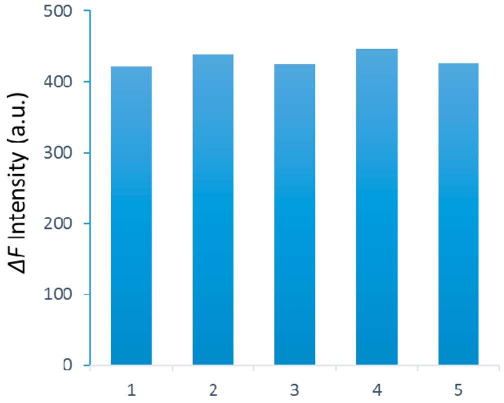
500
400
300
200
100
0
ΔF Intensity (a.u.)
1
2
3
4
5

Fig.S5

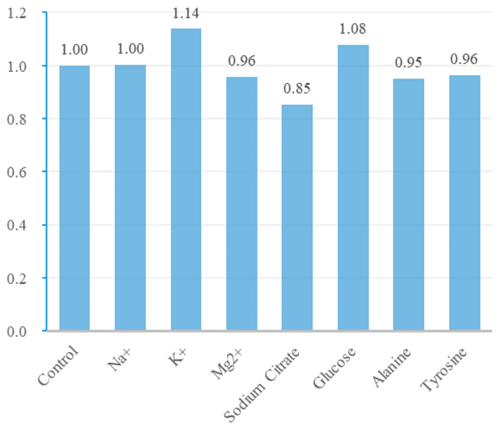

1.00
1.00
1.14
0.96
0.85
1.08
0.95
0.96
1.2
1.0
0.8
0.6
0.4
0.2
0.0
Control
Na+
K+
Mg2+
Sodium Citrate
Glucose
Alanine
Tyrosine

Fig.S6

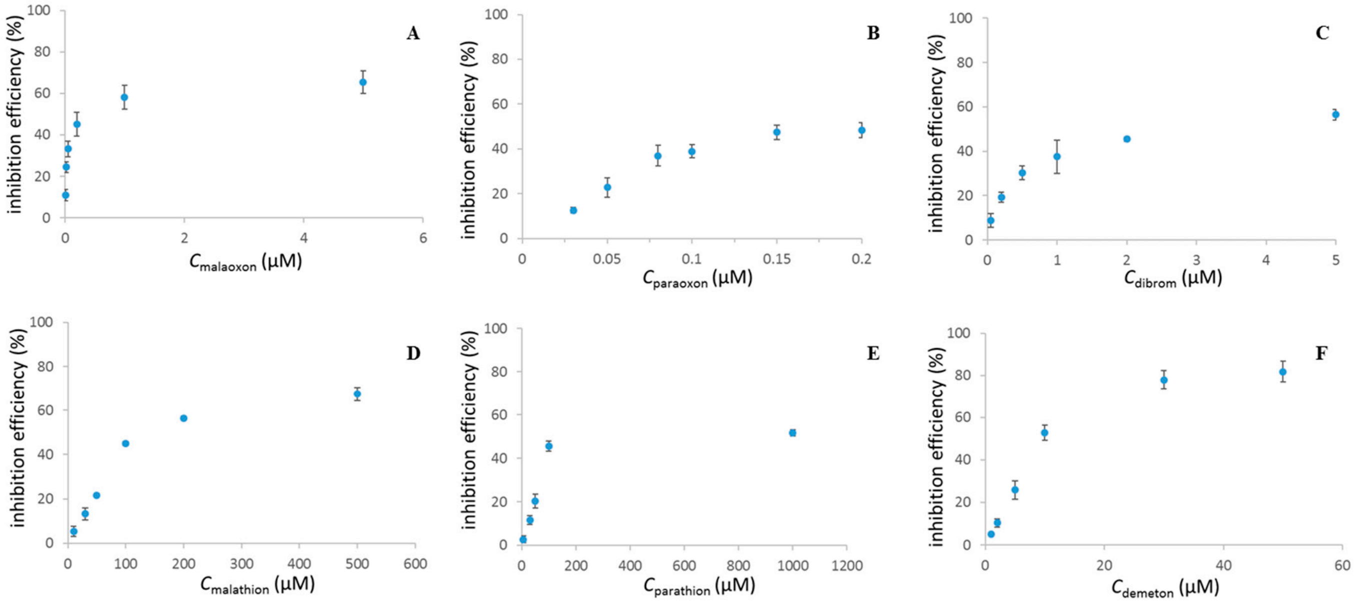

inhibition efficiency (%)
A
$C_{malaoxon}$ (μM)
B
$C_{paraoxon}$ (μM)
C
C_{dibrom} (μM)
D
$C_{malathion}$ (μM)
E
$C_{parathion}$ (μM)
F
$C_{demeton}$ (μM)

Table S1. Recoveries Obtained from the Determination of OPPs in Spiked Sample

Pesticide	Spiked (μM)	Measured (μM)	Recovery (%)
Paraoxon	0	ND[a]	
	0.05	0.05	93.55
	0.2	0.17	84.33
Malaoxon	0	ND	
	0.05	0.07	138.69
	0.2	0.29	142.55
Dibrom	0	ND	
	0.05	0.06	121.35
	0.2	0.16	81.65
Dichlorvos	0	ND	
	0.05	0.04	73.29
	0.2	0.17	85.11
Demeton	0	ND	
	1	0.08	75.83
	5	0.64	106.96
Parathion	0	ND	
	50	46.84	93.68
	100	102.09	102.09
Malathion	0	ND	
	50	34.43	68.87
	100	130.02	130.02
